# Reducing the incidence of Hospital-Acquired Multidrug- Resistant Acinetobacter Baumannii (HA-MDRAB) in the intensive care unit (ICU) of a teaching hospital through multimodal environmental cleaning strategies

**DOI:** 10.1017/ash.2025.113

**Published:** 2025-09-03

**Authors:** Nur Alwani Suhaimi, Haryani Che Hamzah, Masita Ishak, Norzalina Razali, Nur Asyikin Mohd Zaki, Siti Shuhaida Samsudin, Suzana Saaibon, Siti Zuhairah Mohd Razali, Arulvani Rajandra, Nor’azim Mohd Yunos, Sasheela Ponnampalavanar

**Affiliations:** 1 Department of Infection ControlUniversiti Malaya Medical Centre, Kuala Lumpur; 2 Department of Medicine, Faculty of MedicineUniversiti Malaya, Kuala Lumpur; 3 Critical Care Services UnitUniversiti Malaya Medical Centre, Kuala Lumpur

## Abstract

**Introduction:** HA-MDRAB infections and colonizations are known to pose an urgent threat to patients admitted to ICUs worldwide and are difficult to treat, which leads to significant mortality and morbidity. However, multimodal environmental cleaning strategies are effective for MDRAB prevention and control. **Objective:** The objective of this study is to evaluate the impact of multimodal environmental cleaning strategies in reducing HA-MDRAB rate in ICU in a tertiary teaching hospital, Universiti Malaya Medical Centre (UMMC). **Methods:** This is a retrospective analysis of patients admitted to an adult ICU over three periods (P); P1 (January 2018 - December 2019), P2 (January 2020 - June 2022), and P3 (July 2022 – December 2023). The total number of HA-MDRAB infections and colonizers and total patient days in ICU were collected monthly and HA-MDRAB rate per 1000 patient-days was analyzed. The five elements of infection prevention and control (IPC) multimodal strategies involving multidisciplinary teams were integrated with environmental cleaning since 2018, including tailored in-house training for environmental service staff (EVS), cleaning approach, techniques and product used, hospital-wide environmental cleaning policy, use of environmental audit checklist and giving frequent feedback to the EVS, and communication strategies to engage EVS and key stakeholders in order to promote organizational safety culture. **Results:** HA-MDRAB rates in ICU increased by 23% from 7.20 (P1) to 8.85 (P2) per 1000 patient days and decreased to 4.94/1000 patient days (P3) after the reinforcement of environmental cleaning strategies. During P2, the rates were higher from July - December 2021 (15.8/1000 patient days). **Conclusion:** Increase in HA-MDRAB rates was likely due to changes in infection control measures during COVID- 19 pandemic such as extensive workload compromising the compliance to environmental cleaning among EVS. With reinforcement of the environmental cleaning multimodal strategies, HA-MDRAB incidence reduced, emphasizing the importance of adherence to IPC practices in environmental cleaning.

